# Supplementary material for: Overcoming the design, build, test bottleneck for synthesis of nonrepetitive protein-RNA cassettes
Source: Nat Commun. 2021 Mar 11;12:1576. doi: 10.1038/s41467-021-21578-6 (PMC7952577; doi:10.1038/s41467-021-21578-6)
Supplement: Supplementary file 1 — Supplementary Information [file 41467_2021_21578_MOESM1_ESM.docx]

**Supplementary Information**

**Overcoming the design, build, test (DBT) bottleneck for synthesis of protein-RNA binding cassettes**

Noa Katz^1^, Eitamar Tripto^2^, Sarah Goldberg^1^, Orna Atar^1^, Zohar Yakhini^3,4^, Yaron Orenstein^5^, and Roee Amit^1,6^*

^1^ Department of Biotechnology and Food Engineering, Technion - Israel Institute of Technology, Haifa 3200003, Israel.

^2^ Department of Biomedical Engineering, Ben-Gurion University of the Negev, Beer-Sheva 8410501, Israel.

^3^ Department of Computer Science, Technion - Israel Institute of Technology, Haifa 3200003, Israel.

^4^ School of Computer Science, Interdisciplinary Center, Herzliya 46150, Israel.

^5^ School of Electrical and Computer Engineering, Ben-Gurion University of the Negev, Beer-Sheva 8410501, Israel.

^6^ Russell Berrie Nanotechnology Institute, Technion - Israel Institute of Technology, Haifa 3200003, Israel.

Correspondence: [roeeamit@technion.ac.il](mailto:roeeamit@technion.ac.il)

Supplementary information contains 12 Figures, 4 tables, and 7 Movies

#

# Supplementary figures

**Supplementary Figure 1. Histograms of edit distances in the oligo library**


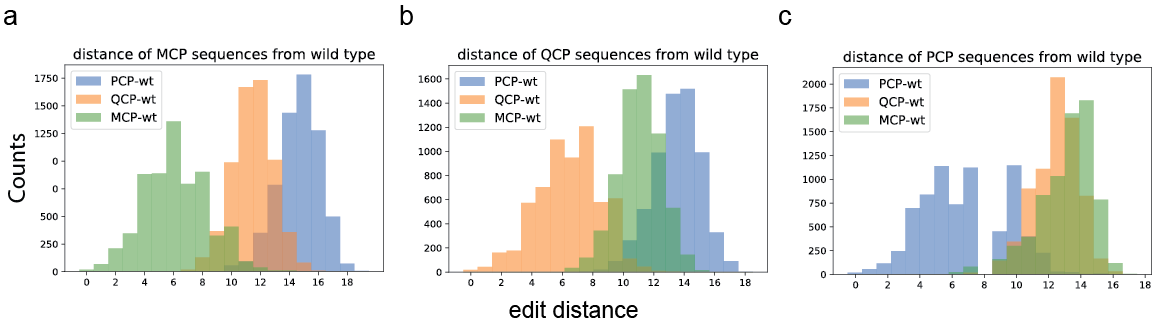


Histograms of the edit distance of the sequences in the library of MCP (a), QCP (b), PCP (c) to the different wild types. The library contains sequences with high similarity to each of the wild types, with larger distances to the wild type of the other proteins.

**Supplementary Figure 2. Flowchart for the preliminary analysis conducted on the reads extracted from the oligo-library experiment**


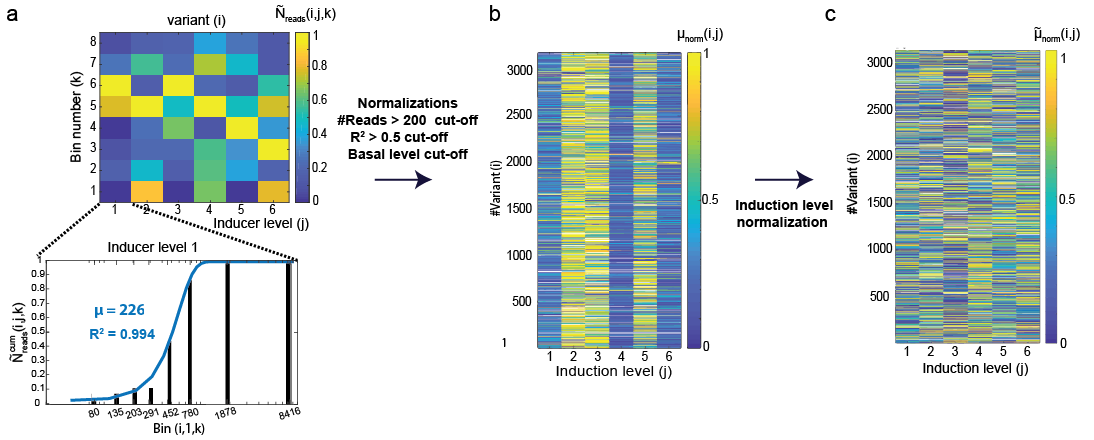


(a) (Top) A sample 6x8 matrix obtained for each variant. (Bottom) Collapsing the matrix to a vector of integrated mCherry level for every inducer value. (b) Sample list for PCP of unsorted non-renormalized 6-long vectors displayed as heatmap. (c) Renormalized heatmap displaying unsorted PCP responsive variants.

**Supplementary Figure 3. Sorted heatmaps for MCP and QCP**


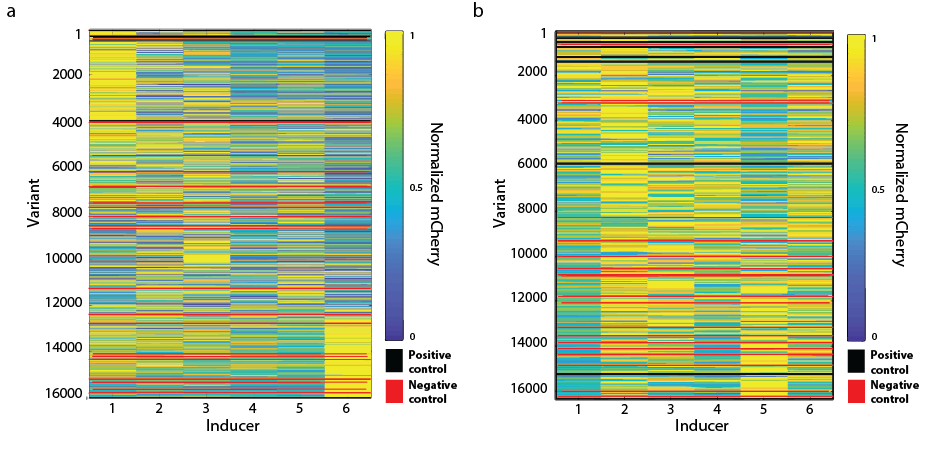


(a) *R_score_* Sorted heat-maps of MCP, and (b) QCP with the OL. Positive and negative control are depicted in black and red, respectively.

**Supplementary Figure 4. Comparison of *R_score_* between C and GC prefixes**

Comparison of the *R_score_* values between C and GC prefixes for the same binding sites of MCP (n=7386) (a), QCP (n=6416) (b), and PCP (n=7339) (c). For all proteins, there is effectively little to no correlation between expression levels and the position of the variants within the ribosomal initiation region.

**Supplementary Figure 5. Comparison between the Gaussian-parametrized *R_score_* computation and the non-parametrized *R_score_* computation**

(Left panels) X-Y scatter plot of the Gaussian-parametrized *R_score_* (X-axis) vs. the non-parametrized R_score_. (Right panels) Cross-correlation computations between the Gaussian-parametrized to the non-parametrized R_score_. The correlation is computed for multiple subsets of variants. Each value on the x-axis corresponds to the last-value on any subset as ordered by the Gaussian-parametrized R_score_. Note, the correlation falls with increasing subset size due to the increased inclusion of non-binders, which are expected to be randomly positioned in both the parametrized and non-parametrized spaces.

**Supplementary Figure 6. Comparison of structure-conserving ML mutation analysis for the non-parametrized (left panels) vs. the Gaussian-parametrized (right panels) approach**

**
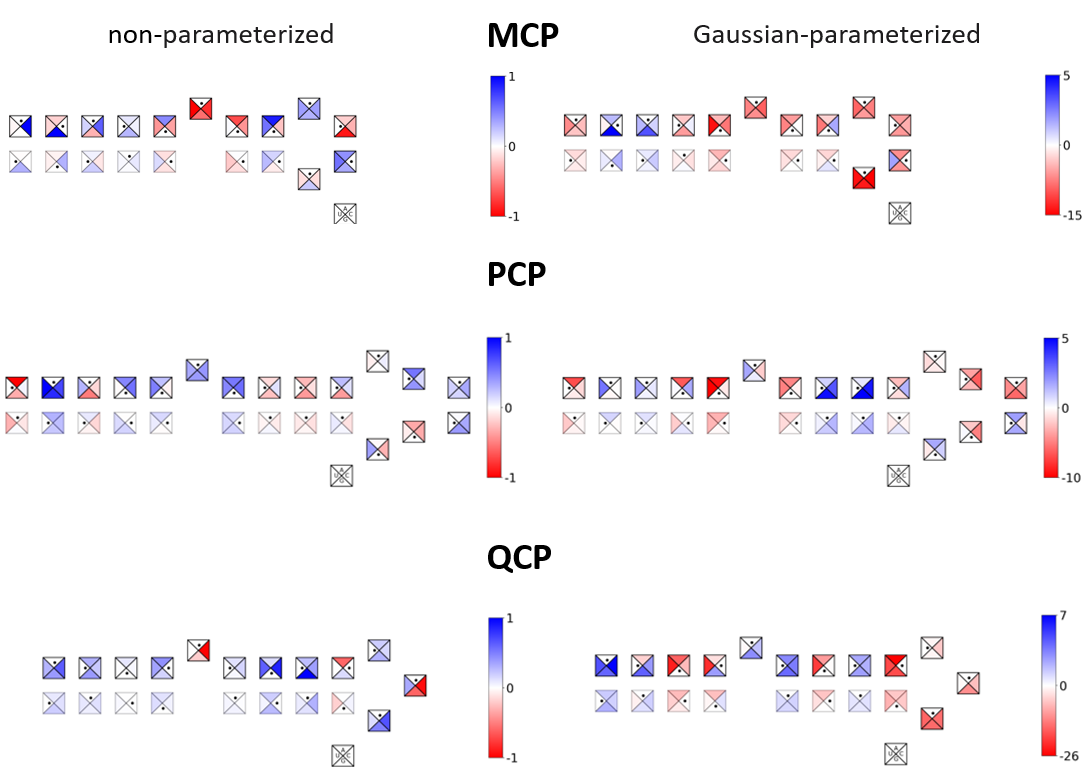
**

**Supplementary Figure 7.** **Performance evaluation of the whole-library models with the structural contribution**


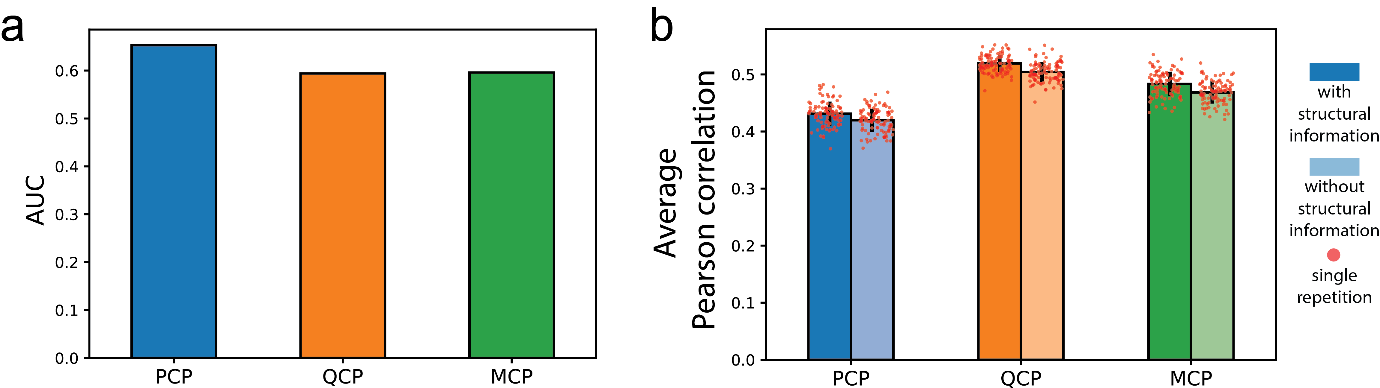


Performance evaluation of the whole-library model in binary classification and by including RNA structural information. (a) Whole-library model performance reported in AUC on a held-out test set. (b) Average and standard deviation of Pearson correlation achieved over 100 iterations of training and testing the whole-library model on random partitions of the data into training and test sets with and without structural information. The results show that for all cases when the model was trained with structural information, its performance improved compared to models trained without structural information (p-values= 3∙10^-10^, 5∙10^-7^, 3.5∙10^-14^ for MS2, PP7 and Qb, respectively. Two sided paired Wilcoxon rank-sum test, n=100).

**Supplementary Figure 8.** **Negative controls for fluorescent experiments in U2OS cells**


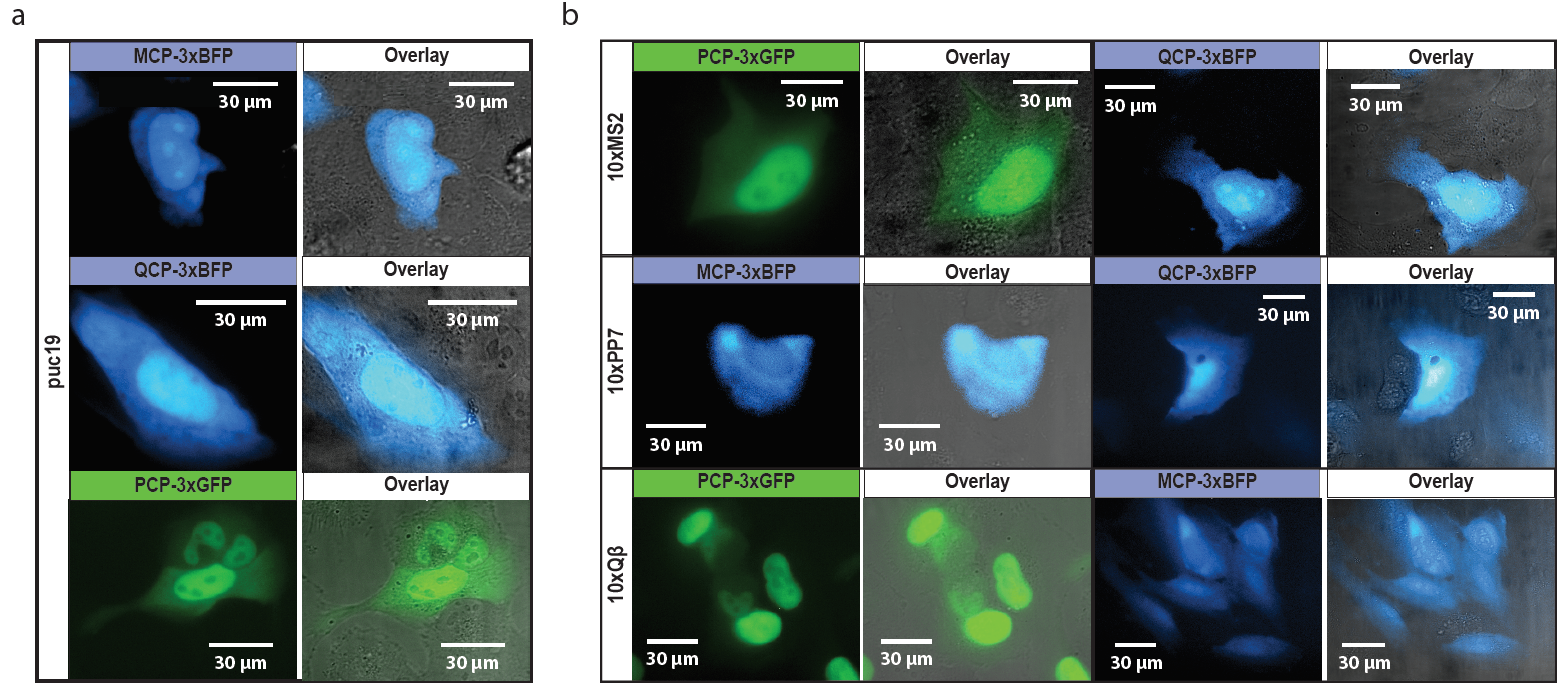


(a) Microscopy images of RBP-3xFP with plasmid containing no binding sites cassettes (puc19). (b) Additional negative control images, where RBP-3xFP plasmids were transfected with non-cognate cassettes. For each experiment, both the relevant fluorescent channel and the merged images with the differential interference contrast (DIC) channel are presented, and fluorescent wavelengths used in these experiments were: 400nm for BFP and 490nm for GFP. For both panels, no fluorescent *foci* were detected. Each experiment was successfully conducted in duplicates and on two different days.

**Supplementary Figure 9.** **PCP with 10xPP7 model based**

Microscopy images of PCP-3xBFP with a cassette containing binding sites predicted by the ML model. Both the relevant fluorescent channel and the merged images with the differential interference contrast (DIC) channel are presented, and the fluorescent wavelength used was 490nm. This experiment was successfully conducted in duplicates and on two different days.

**Supplementary Figure 10. QQ-plot computation for the *R_score_* of positive and negative controls**

Positive(left) and negative (right) controls for (top) PCP, (middle) MCP, and (bottom) QCP.

**Supplementary Figure 11. Conversion of *R_score_* to K_d_**


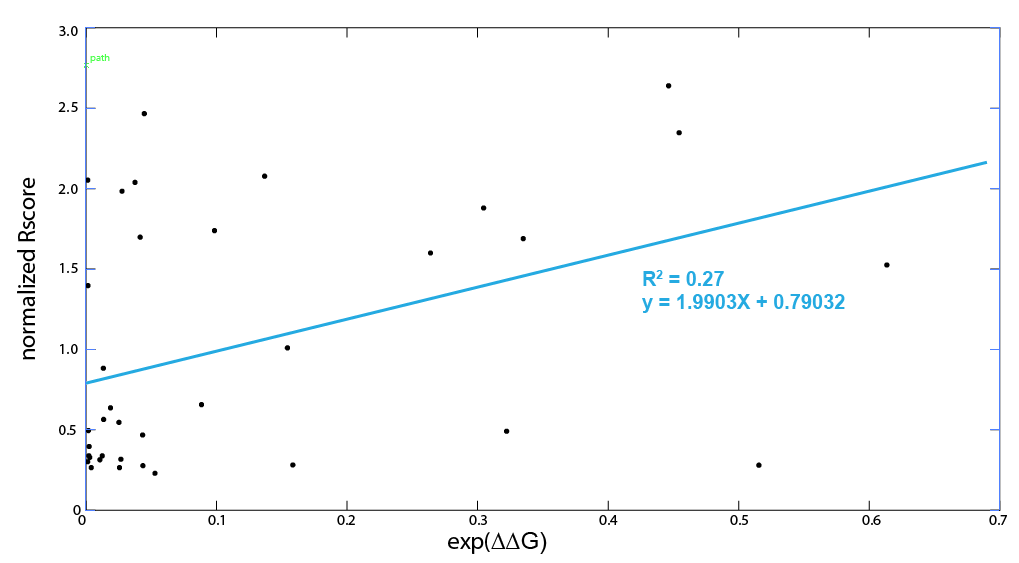


Experimental normalized *R_score_* as a function of ΔΔG results of a previous study for 37 mutual binding sites^17^. Only binding sites with measurable affinity- R_score_ (>3.5) and ΔΔG (>-6.66169) are taken into account. The linear regression results are presented in blue along with its goodness of fit (R^2^).

**Supplementary Figure 12. Illustration of the hyper-parameters search, model training and evaluation processes**


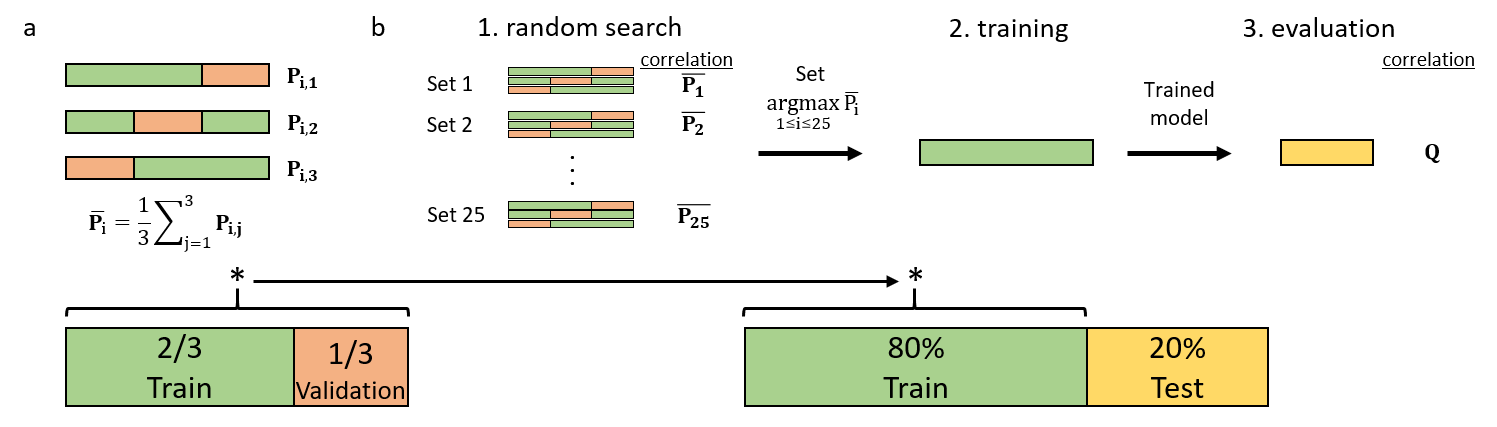
(a) 3-fold cross validation (CV) process. The training set (80% of the data) is divided into 3 folds. In iteration j for j=1,2,3, fold j is used as a validation fold, and the two other folds are used for training. (b) (left to right) Stage 1 – repeating 25 times: randomly selecting hyper-parameters and performing 3-fold CV on the training set. Stage 2 – selecting the set of parameters from stage 1 achieving maximum average Pearson correlation, and training the model using the data used in stage 1 (80% of the data). Stage 3 - evaluating the model on a held-out test set (remaining 20% of the data).

**Supplementary Figure 13. Raw FACS output file demonstrating the gating strategy**


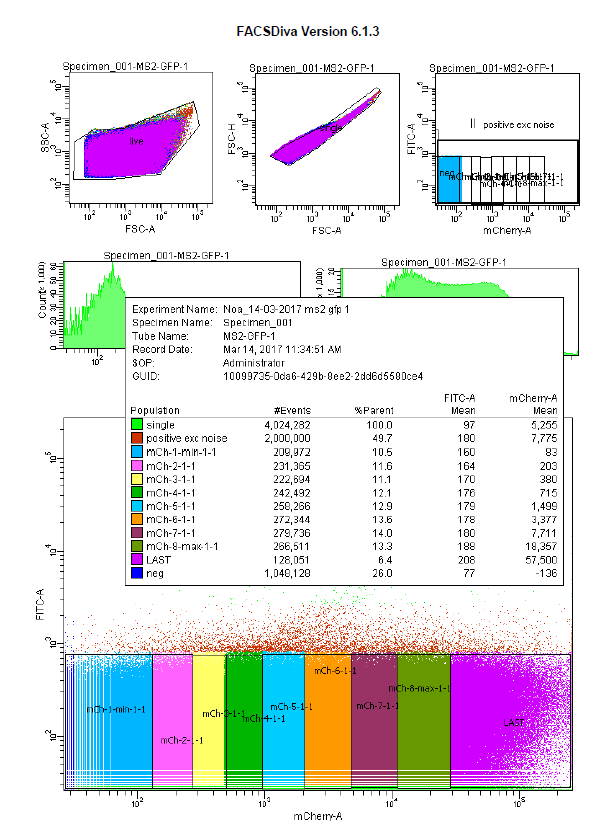


**c**

**b**

**a**

(a) Example graphs representing the gating strategy on the SSC-A and FSC-A parameters during cell sorting for a sorting experiment. (left) Live cells gating, (middle) single cell gating, and (right) illustration of the mCherry bins distribution. (b) A plot of the FITC-A vs. mCherry-A data for a single sorting experiment, going from low mCherry levels in bin number one (left-blue) to high mCherry levels for bin number eight (right-green). (c) Statistics for the example sorting experiment. Starting from single cells (bright green) to each of the mCherry bins (blue to green).

**Supplementary Movie Legends:**

**Supplementary Movie 1.** MCP-BFP + 10xMS2-OL

**Supplementary Movie 2.** PCP-GFP + 10xPP7-OL

**Supplementary Movie 3.** MCP-BFP + QCP-mCherry + 10xMS2-OL + 10xQB-OL

**Supplementary Movie 4.** PCP-GFP + 10xQβ-PP7 model

**Supplementary Movie 5.** QCP-BFP + 10xQβ-PP7 model

**Supplementary Movie 6.** QCP-BFP + 10xMS2-noWT (negative control)

**Supplementary Movie 7.** PCP-GFP + 10xPP7 model
