# Supplementary material for: Overcoming the design, build, test bottleneck for synthesis of nonrepetitive protein-RNA cassettes
Source: Nat Commun. 2021 Mar 11;12:1576. doi: 10.1038/s41467-021-21578-6 (PMC7952577; doi:10.1038/s41467-021-21578-6)
Supplement: Supplementary file 15 — Reporting Summary [file 41467_2021_21578_MOESM15_ESM.pdf]

nature research

Corresponding author(s): Roee Amit

Last updated by author(s): Dec 17, 2020

Reporting Summary

Nature Research wishes to improve the reproducibility of the work that we publish. This form provides structure for consistency and transparency in reporting. For further information on Nature Research policies, see our Editorial Policies and the Editorial Policy Checklist.

Statistics

For all statistical analyses, confirm that the following items are present in the figure legend, table legend, main text, or Methods section.

n/a	Confirmed	
☐	☒	The exact sample size (n) for each experimental group/condition, given as a discrete number and unit of measurement
☐	☒	A statement on whether measurements were taken from distinct samples or whether the same sample was measured repeatedly
☐	☒	The statistical test(s) used AND whether they are one- or two-sided<br>*Only common tests should be described solely by name; describe more complex techniques in the Methods section.*
☒	☐	A description of all covariates tested
☒	☐	A description of any assumptions or corrections, such as tests of normality and adjustment for multiple comparisons
☐	☒	A full description of the statistical parameters including central tendency (e.g. means) or other basic estimates (e.g. regression coefficient) AND variation (e.g. standard deviation) or associated estimates of uncertainty (e.g. confidence intervals)
☒	☐	For null hypothesis testing, the test statistic (e.g. F, t, r) with confidence intervals, effect sizes, degrees of freedom and P value noted<br>*Give P values as exact values whenever suitable.*
☒	☐	For Bayesian analysis, information on the choice of priors and Markov chain Monte Carlo settings
☒	☐	For hierarchical and complex designs, identification of the appropriate level for tests and full reporting of outcomes
☐	☒	Estimates of effect sizes (e.g. Cohen's d, Pearson's r), indicating how they were calculated

Our web collection on statistics for biologists contains articles on many of the points above.

Software and code

Policy information about availability of computer code

Data collection	FACS data was collected using BD FACSDiva Software version 8.0.1, firmware version 1.8.<br>Illumina sequencing data was collected using Hiseq Control Software version 2.2.70, library version 3.8.358.
Data analysis	Matlab software was used for variant read counting, linear regression, and responsiveness analysis. Python was used all the learning algorithms. Vienna package was used for RNA structure prediction. Code available at: https://github.com/OrensteinLab/SynRBPbind

For manuscripts utilizing custom algorithms or software that are central to the research but not yet described in published literature, software must be made available to editors and reviewers. We strongly encourage code deposition in a community repository (e.g. GitHub). See the Nature Research guidelines for submitting code & software for further information.

Data

Policy information about availability of data

All manuscripts must include a data availability statement. This statement should provide the following information, where applicable:

- Accession codes, unique identifiers, or web links for publicly available datasets
- A list of figures that have associated raw data
- A description of any restrictions on data availability

Processed data is available at https://github.com/OrensteinLab/SynRBPbind.

Field-specific reporting

Please select the one below that is the best fit for your research. If you are not sure, read the appropriate sections before making your selection.

☒ Life sciences ☐ Behavioural & social sciences ☐ Ecological, evolutionary & environmental sciences

For a reference copy of the document with all sections, see nature.com/documents/nr-reporting-summary-flat.pdf

Life sciences study design

All studies must disclose on these points even when the disclosure is negative.

Sample size	Sample size for the flow cytometry was chosen based on the number of variants in the library. There are 20,000 different variants in the library. 300,000 cells per expression bin were collected to enable coverage of all library variants, while allowing for an order of magnitude variation between the frequencies of the different library variants.
Data exclusions	We conducted data exclusion procedure on the oligo-ibrary strains based on their read numbers following FACS sorting and NGS. The following two criteria were predetermined based on previous works. We introduced two cutoffs on the 8x6 (expression x induction) matrices for each of the different variants: (i) only inducer levels that had above 30 reads for all eight expression bins combined were taken into account; and (ii) only variants that had more than 300 reads in total for the entire 8x6 matrix were taken into account.
Replication	For the oligo-library work we had six different inducer concentrations, yet no duplicates for each one. This was due both to workload and to the financial expense of the entire protocol. As for the imaging experiments, each experiment was done in duplicates and on at least two different days (total of 4 experiments per variant tested); all attempts at replication were successful.
Randomization	During the oligo-library sorting, all six samples that had different levels of inducer concentrations were allocated to one of two consecutive days, i.e. inducer levels zero, three, and five were done on day one, and levels one, two, and four on day two.
Blinding	Blinding was not relevant to the study, since the analysis for all samples was identical and automated.

Reporting for specific materials, systems and methods

We require information from authors about some types of materials, experimental systems and methods used in many studies. Here, indicate whether each material, system or method listed is relevant to your study. If you are not sure if a list item applies to your research, read the appropriate section before selecting a response.

Materials & experimental systems

n/a	Involved in the study	
☒	☐	Antibodies
☐	☒	Eukaryotic cell lines
☒	☐	Palaeontology and archaeology
☒	☐	Animals and other organisms
☒	☐	Human research participants
☒	☐	Clinical data
☒	☐	Dual use research of concern

Methods

n/a	Involved in the study	
☒	☐	ChIP-seq
☐	☒	Flow cytometry
☒	☐	MRI-based neuroimaging

Eukaryotic cell lines

Policy information about cell lines

Cell line source(s)	Human Bone Osteosarcoma Epithelial Cell line (U2OS, HTB-96 in ATCC)
Authentication	None of the cell lines were authenticated.
Mycoplasma contamination	Cell lines were tested for mycoplasma contamination and were found negative.
Commonly misidentified lines (See ICLAC register)	No commonly misidentified cell lines were used in the study.

Flow Cytometry

Plots

Confirm that:

- [x] The axis labels state the marker and fluorochrome used (e.g. CD4-FITC).
- [x] The axis scales are clearly visible. Include numbers along axes only for bottom left plot of group (a 'group' is an analysis of identical markers).
- [x] All plots are contour plots with outliers or pseudocolor plots.
- [x] A numerical value for number of cells or percentage (with statistics) is provided.

Methodology

Sample preparation	Each sample had prior to sorting: E.coli Top10 cells (Invitrogen, C404006) that were shaken at 250rpm and 37 degrees in semi-poor medium (see Methods) containing different concentrations of N-butanoyl-L-homoserine lactone (C4-HSL, Cayman Chemical K40982552 019) for three hours. When sorting multiple experimental conditions in the same day, cells were kept on ice.
Instrument	BD Biosciences FACSAria IIIu
Software	BD FACSDiva Software version 8.0.1 (build 2014 07 03 11 47), Firmware version 1.8 (BD FACSAria II), CST version 3.0.1, PLA version 2.0
Cell population abundance	Post-sort fractions contained only bacterial cells cells with the chosen levels of GFP and mCherry. 300,000 cells were collected per bin.
Gating strategy	Live and single (as opposed to doublet) cells were gated based on the FSC-SSC scatter plot.

- [x] Tick this box to confirm that a figure exemplifying the gating strategy is provided in the Supplementary Information.
