# Supplementary material for: Overcoming the design, build, test bottleneck for synthesis of nonrepetitive protein-RNA cassettes
Source: Nat Commun. 2021 Mar 11;12:1576. doi: 10.1038/s41467-021-21578-6 (PMC7952577; doi:10.1038/s41467-021-21578-6)
Supplement: Supplementary file 16 — Description of Additional Supplementary Files [file 41467_2021_21578_MOESM16_ESM.pdf]

Title: Supplementary Data 1:
Description: A list of the OL variant sequences. For each variant the list provides: variant name, variant sequence, predicted structure, computed R_{score}, and computed ΔΔG. A separate list is provided for each CP used in the experiment (MCP, QCP, and PCP).

Title: Supplementary Data 2:
Description: A list of all primers used in the various experiments.

Title: Supplementary Data 3:
Description: Sequences used for the coat-proteins in the experiment.

Title: Supplementary Data 4:
Description: Sequences used for encoding the various RNA cassettes used for validation experiments in both bacteria and mammalian cells.

Title: Supplementary Data 5:
Description: List of all barcodes used in the oligo library to label the different variants.

Title: Supplementary Movie 1.
Description: MCP-BFP + 10xMS2-OL

Title: Supplementary Movie 2.
Description: PCP-GFP + 10xPP7-OL

Title: Supplementary Movie 3.
Description: MCP-BFP + QCP-mCherry + 10xMS2-OL + 10xQB-OL

Title: Supplementary Movie 4.
Description: PCP-GFP + 10xQβ-PP7 model

Title: Supplementary Movie 5.
Description: QCP-BFP + 10xQβ-PP7 model

Title: Supplementary Movie 6.
Description: QCP-BFP + 10xMS2-noWT (negative control)

Title: Supplementary Movie 7.
Description: PCP-GFP + 10xPP7 model
